# Population Size Estimation of Men Who Have Sex with Men in Tbilisi, Georgia; Multiple Methods and Triangulation of Findings

**DOI:** 10.1371/journal.pone.0147413

**Published:** 2016-02-01

**Authors:** Lela Sulaberidze, Ali Mirzazadeh, Ivdity Chikovani, Natia Shengelia, Nino Tsereteli, George Gotsadze

**Affiliations:** 1 Curatio International Foundation, Tbilisi, Georgia; 2 Global Health Sciences, University of California San Francisco, San Francisco, California, United States of America; 3 Regional Knowledge Hub, and WHO Collaborating Centre for HIV Surveillance, Institute for Futures Studies in Health, Kerman University of Medical Sciences, Kerman, Iran; 4 Center for Information and Counseling on Reproductive Health–Tanadgoma, Tbilisi, Georgia; National Center for AIDS/STD Control and Prevention, China CDC, CHINA

## Abstract

**Introduction:**

An accurate estimation of the population size of men who have sex with men (MSM) is critical to the success of HIV program planning and to monitoring of the response to epidemic as a whole, but is quite often missing. In this study, our aim was to estimate the population size of MSM in Tbilisi, Georgia and compare it with other estimates in the region.

**Methods:**

In the absence of a gold standard for estimating the population size of MSM, this study reports a range of methods, including network scale-up, mobile/web apps multiplier, service and unique object multiplier, network-based capture-recapture, Handcock RDS-based and Wisdom of Crowds methods. To apply all these methods, two surveys were conducted: first, a household survey among 1,015 adults from the general population, and second, a respondent driven sample of 210 MSM. We also conducted a literature review of MSM size estimation in Eastern European and Central Asian countries.

**Results:**

The median population size of MSM generated from all previously mentioned methods was estimated to be 5,100 (95% Confidence Interval (*CI*): 3,243 ~ 9,088). This corresponds to 1.42% (95%*CI*: 0.9% ~ 2.53%) of the adult male population in Tbilisi.

**Conclusion:**

Our size estimates of the MSM population (1.42% (95%*CI*: 0.9% ~ 2.53%) of the adult male population in Tbilisi) fall within ranges reported in other Eastern European and Central Asian countries. These estimates can provide valuable information for country level HIV prevention program planning and evaluation. Furthermore, we believe, that our results will narrow the gap in data availability on the estimates of the population size of MSM in the region.

## Introduction

Men who have sex with men (MSM) have a greater risk of HIV infection than the general population and are approximately 19 times more likely to be living with HIV [1]. In Georgia, HIV prevalence within at-risk groups is reported to be the highest amongst MSM. In 2012, the HIV prevalence was reported to be 13% with an increasing trend over recent years [2].

Given the limited local and international resources available for controlling the HIV epidemic, it is necessary to advocate for the most effective prevention/intervention strategies within key populations at high-risk of HIV. Accurate estimate of the number of MSM is crucial to advocacy, resource allocation, intervention planning, program monitoring and evaluation.

Due to the stigma of same sex sexual behavior, measuring this hard to reach population continues to be a challenge in the region. Homosexuality was decriminalized in Georgia in 2000, however, a significant level of stigma and discrimination persist throughout the country, including urban areas such as Tbilisi. This stigma makes it challenging to identify MSM and accurately calculate the impact of the epidemic in the region.

A methodological challenge also exists: there is currently no widely recognized gold standard for population size estimation (PSE) of a hidden and hard to reach community like MSM. In the absence of a gold standard, estimate from one method is empirically imprecise and prone to potential biases. This comparative study utilized a number of well-known and innovative methods to provide a range of estimates for a population size of MSM in Tbilisi, the capital city of Georgia. We applied several methods: literature review, network scale-up, mobile/web apps multiplier, service and unique object multiplier, network-based capture-recapture, Handcock RDS-based and Wisdom of Crowds to estimate the size of the MSM population in Tbilisi, Georgia.

## Methods

In addition to literature review, we integrated six population size estimation methods into two surveys during in Tbilisi, Georgia in 2014. The first was a household survey administered to 1,015 adult individuals. Data from this survey was used in a Network Scale-Up Method (NSUM) of PSE. Another survey was conducted among 210 MSM recruited through Respondent Driven Sampling (RDS). This survey was used to measure popularity and transparency biases so that NSUM findings could be adjusted. Other size estimation methods were also applied to the RDS survey data. Below are descriptions of each of the PSE methods implemented in this study:

### Network Scale-Up Method (NSUM)

The general concept behind network scale-up method is that an individual’s social network is representative of the whole population. That is, one person’s group of friends reflects the characteristics of the community as a whole. By asking the respondent questions about an acquaintance, rather than the respondent themselves, the interview takes on some anonymity allowing the responses to be honest without fear of stigma or other negative consequences [3–7].

Using cluster random sampling, in a household survey, we recruited 1,015 adults (18 years old or greater) in Tbilisi to estimate the network size and the size of MSM population. As for the sampling issues a two-stage stratified sampling was used. The National Statistics Department election list for 2010 was used as a sampling frame. Tbilisi is divided by municipalities (strata) and election areas. The latter were selected as primary sampling units (PSU) and households as the second. Number of households in each PSU was defined as five. Within each municipality, the number of PSUs were selected based on the probability proportion to size method. PSUs were randomly selected from the list. Within each PSU, the random walk method was used to select households. Within each selected household, one person aged 18–49 years was selected for interview (based on last birthday). If there were no response at the household after 3 visits (on different days and different times) the next household was selected.

In a face-to-face interview, we asked the recruited subjects about the number of men they knew who had sex with other men in the last year. We clarified first that by “knowing” we meant, the subject could identify the person by face or name and be able to contact them if they should wish to. We also asked them about the number of people they know from the 24 groups with “known size” population to estimate the social network size (more details below).

In NSUM, we need three parameters to estimate the population size of the target group:

The average social network size of respondent i = c_i_
Number of people from the target group who are known to the respondent i = m_i_
The total adult (>18 years old) population of Tbilisi = t

Using the maximum likelihood estimator proposed by Killworth et al. [8] the population size estimation is equal to

$${\text{PSE}}_{\;(\text{Network Scale-Up})}=\;e=\frac{\sum_{i}m_{i}}{\sum_{i}\hat{c}}\;t$$ (Eq 1)

To estimate the social network size, we applied the known-size population approach. We used 24 known size populations (j = 24), to back calculate the average social network size. Calculations were made using the following steps:

1. Solve the Eq 1 to estimate the network size for every respondent (i) using all eligible populations with known size (j):
  $$c_{i}=\frac{\sum_{ij}m_{ij}}{\sum_{j}e_{j}}\;t$$ (Eq 2)
2. Make the average of C_i_ and use the average ($\hat{c}$) to back calculate the size of every populations:
  $$e_{j}=\frac{\sum_{ij}m_{ij}}{\sum_{i}\hat{c}}\;t$$ (Eq 3)
3. Devide the estimated size (e) by the real size (E) of each 24 population with known size to measure the bias factor:
  $${Bias\;factor}_{i}=\frac{E_{i}}{e_{i}}$$ (Eq 4)
4. If any of the bias factors are more than 1.5 or less than 0.5, drop the population with the most deviance. Go to step 1, and repeat the process.
5. Stop when all bias factors are within the range of 0.5 to 1.5 and report the average social network size.

After applying this process, we ended up with 21 eligible populations. Now, given all parameters in the Eq 1, we calculated the size of the MSM population. The variance of the estimated population size was calculated using bootstrap simulation.

In order to adjust the NSUM estimates for its two known biases, information transparency bias (MSM may not openly talk to others about their sexual orientation or behaviors) and popularity ratio (in comparison to others, MSM may have smaller network sizes and therefore are less likely to be counted in social networks), 210 MSM who provided verbal informed consent and agreed to participate in the study, were recruited by the RDS method through peer-referrals initiated with 10 seeds. We selected seeds based on age ranges (18–30 or >30 years old), geographic areas (5 main districts), socio-economic status (low-middle or high) and places where sex partners are sought (bars or other public areas, or through the Internet). Each was asked to recruit 3 eligible MSM. Respondents were given 15 USD for participating and 3 USD for every successfully recruited peer. Men who have self-reported having sex with another men during the last 12 month prior to the interview, aged 18 years or older, living in Tbilisi during the period of study and provided informed consent were eligible and recruited in the survey.

### Multiplier Methods

In the MSM RDS survey, we integrated several methods to estimate the size of the MSM population, collectively known as “multiplier methods”. Three different types of multipliers were used: Service Multiplier [9], Unique Object Multiplier [10], and Web/Mobile Apps Multiplier [11]. The Service Multiplier involved the use of programmatic data from a health center, which was cross-referenced with data collected from respondents about the utilization of specific services over the six months prior to the survey. We collected the number of MSM who used some specific services over the six months prior to the survey. In the Unique Object Multiplier, 500 leather bracelets (as unique objects) were distributed amongst eligible MSM by outreach workers prior to the RDS survey. The Web/Mobile Apps Multiplier assessed the utilization of the most popular websites (Mamba.ru, Gayromeo.com) and mobile phone applications (Grindr and Hornet) among the Georgian MSM population. Counts of MSM (duplicates removed) were obtained using mobile and web apps over the course of two weeks prior to the interview and three weeks during the survey. In the MSM RDS survey, we asked the participants, whether they had been given unique objects, had received specific health services at the center or had used one of the mobile applications or websites over the specified time period. Since the recruitment was based on peer-chain referrals and seeds were selected with a non-random purposeful process, RDS Analyst Software [12] was used to estimate the proportion of MSM who have received specific health services, unique object or used the mobile apps/website. These two different sources of data were used to estimate the population size of MSM in Tbilisi. In the multiplier method, we need two parameters to estimate the population size:

Number of MSM who have received the health services = n
The proportion of MSM in the RDS survey that have reported receiving such services = p

And the size can be estimated using the following equation:

$${\text{PSE}}_{\;(\text{multiplier})}=e=\frac{n}{p}$$ (Eq 5)

The calculations for unique object or mobile apps/website user multiplier, the calculation is the same as explained above, but the sources of data are different. The variance of the estimated population size was calculated with Delta Method [9].

### Network based Capture-Recapture

Another method that we integrated into the RDS survey was network-based capture-recapture, a novel method proposed by Dombrowski [13]. In this method study participants were asked to provide their own personal information (height, approximate weight, hair color and ethnicity) and “telefunken code”, which was derived from the last four digits of their own mobile phone number. The code is created where phone digits are coded as odd or even and low (0–4) or high (5–9). Following completion of the survey the participants’ personal information and “telefunken code” were recorded. The respondents were then asked to randomly select five MSM contacts from their mobile phone directory. For respondents with five or less MSM contacts all of the contacts were selected. The respondent was then questioned about the randomly selected contacts, in order to obtain data on the contacts’ personal characteristics and “telefunken code”. The coded phone number (telefunken) together with the height, approximate weight, hair color, and ethnicity produced (almost) a unique anonym code for each respondent that facilitated the matching of the respondent to contacts described by another respondent interviews. For the purposes of population estimate, study participants were treated as the “capture” population, while each of the contacts provided during the interviews (“reports”) were considered a “recapture assay”. Given the number of the original respondents discovered via recapture assays (as a proportion of the total number of assays), a basis was established for estimating the overall size of the MSM population. Here is the required parameters and formula on how to estimate the population size:

Number of MSM captured in the survey with valid telefunken codes = n
Number of valid telefunken reported by MSM in the study = s
Excluding false matches, number of MSM’s telefunken that mentioned by other MSM = r

$${\text{PSE}}_{\;(\text{Network based Capture-Recapture})}=e=\frac{n\times s}{r}$$ (Eq 6)

We applied the following formula to estimate the standard error for the population size:

$${\text{SE}}_{\text{e}}=\sqrt{\frac{\text{n}\times \text{s}\times (\text{n}-\text{r})\times (\text{s}-\text{r})}{{\text{r}}^{3}}}$$ (Eq 7)

### Handcock’s Respondent Driven Sampling (RDS) based Method

Handcock’s RDS based method uses a successive sampling approximation to the RDS to leverage information in the ordered sequence of observed personal network sizes. The inference uses the Bayesian framework [14], allowing for the incorporation of prior knowledge to make inferences about population size 24. In our study, estimates were used from the NSUM as prior estimate on the size of MSM in Tbilisi. Given such prior knowledge and the likelihood of observed successive decrease in degree of recruited participants in the MSM RDS survey, we developed the posterior distribution of MSM population size in Tbilisi. The calculation was done using RDS Analyst Software (version 0.42).

### Wisdom of the Crowds

Participants in the RDS survey were asked for their best guess on the number and range of the population size of MSM in Tbilisi, as an application of the Wisdom of the Crowds [15]. This method is based on the assumption that, in aggregate, the responses of sufficient number of MSM population on their numbers will provide a good estimate of the actual number of their population [16;17]. In the RDS survey, we asked the recruited MSM about their own believes on the overall and range of MSM population size in Tbilisi. Later, we calculated the median for the point, minimum and maximum number of MSM reported by the study participants.

As described, multiple size estimation methods were applied to improve the accuracy of the final estimate of MSM population size as well as providing upper and lower acceptability bounds so as to reduce the likelihood that biases of any single method would substantially alter results.

For the household survey, we calculated the sample size for estimating the social network size. Using the range for the social network size of 303 for Iranian population [18] and 251 for people living in Rwanda [19], following our discussion within our research team, we agreed that 300 can be a good approximation for the network size of people living in Georgia. Assuming a Poisson distribution for social network size, such distribution can be approximated by a normal distribution with mean of λ (= 300) and standard error as square root of λ (= 17.3). Given the response rate of 90%, margin of error as 10% of the standard error, and design effect of 3, the total sample size was calculated as 1281 for the whole country of Georgia. For Tbilisi with the population of 1,158,000, we considered 1,000 as the sufficient minimum sample size.

For the MSM survey, the sample size was calculated for the purpose of estimating the social network size of MSM. Assuming the design effect of 1.1 [20], the average social network size of 270 for the MSM population [18] with a Poisson distribution which approximated by a normal distribution with mean of 270 and standard error of 16.4 (as explained above), the margin of error as 15% of the overall standard error, and the confidence level of 95%, we came up with the minimum sufficient sample size of 209 for the RDS study.

The study protocol and procedures were reviewed and approved by the Ethical Committee of the HIV/AIDS Patients Support Foundation (03/28/2014—Certificate N719/820). Verbal informed consent was obtained from each participant prior to the interview.

Before data collection, all the interviewers were trained in discussing sensitive issues and protecting participants’ confidentiality and human rights. The NSUM questionnaire was first developed in English then translated into Georgian and piloted among 20 Household members in three different districts of Tbilisi. Feedback and expert input following the pilot phase provided an opportunity to modify and improve the final version of the questionnaire. The questionnaire collected information about the demographics of the respondent, number of acquaintances they know from each 24 known-size population and number of MSM they know among their friends. The data collection form in the RDS survey was also validated using expert opinion and a pilot phase having 10 MSM participated in 2 focus group discussions.

**Fig 1:**
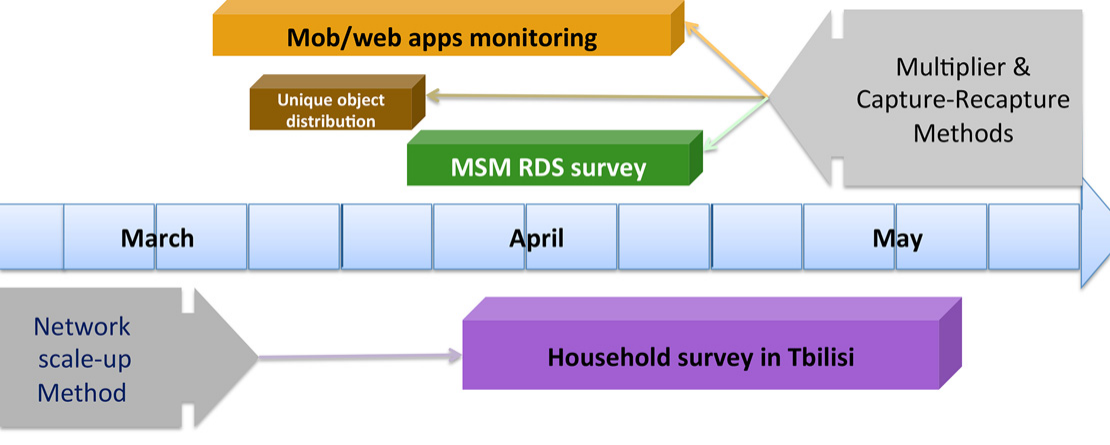
MSM population size estimation study timeline.

The implementation took place between March and May 2014. The timeline of the study is presented below (Fig 1).

In addition to the multiple PSE methods a literature review was undertaken to provide an overview of the estimates of MSM from other Eastern European and Central Asian (EECA) countries for comparison. Review was conducted through searches in PubMed with combination of the following key terms: men who have sex with men, population size estimation, prevalence estimates and country names of EECA region. English language articles published between 2005 and 2015 were reviewed. Google and UNAIDS webpages were screened for a grey literature like country reports and/or for other materials. The results table, which consolidates the PSE data found by literature review, is presented in the discussion.

## Results

### Overall findings

The estimated MSM population sizes using different methods are presented in Table 1. Taking into account all different PSE methods, the median estimates for the size of MSM population are 5,100 (95% (*CI*): 3,243 ~ 9,088). This corresponds to 1.42% (95% (*CI*): 0.9% ~ 2.53%) of adult male population in Tbilisi. As part of sensitivity analysis, when we excluded the WOC estimates from the combined population size, the point estimate of the population size (the median of all methods excluding the WOC) decreased to 4,385 (95% (CI), 3,115 ~ 8,759).

**Table 1:** Different MSM population size estimates from various methods implemented in Tbilisi, Georgia 2014.

Various PSE methods	Point estimate		Lower Bound		Upper Bound	
	18–59y	Total	18–59y	Total	18–59y	Total
Network Scale-up	5816	6014	4972	5197	6859	7176
Multipliers						
*Grindr*	9636		5701		31097	
*Mamba*	5881		3372		22961	
*Hornet*	22859		11362		22859	
*Gayromeo*	3201		2029		7589	
Service utilization	1980		1116		8759	
Unique object	988		607		2648	
Network based Capture-Recapture	4385		3115		5654	
Handcock RDS-based method	2665		344		9417	
Wisdom of the Crowds	15000		5000		30000	
**MSM size—Median of all above estimates**	**5100**		**3243**		**9088**	
**MSM prevalence in adult population**	**1.42%**		**0.90%**		**2.53%**	

The unique object multiplier yielded to the lower as 988 (acceptable interval 607–2648), while using *Hornet* as the multiplier estimates was 22,859. NSUM estimates were the most precise estimates with the smallest range (1887) compared to the range of *Grindr’s* estimates (25,396) and Wisdom of Crowd’s estimates (25,000).

### Active social network size

To calculate the average size of an active social network, we used a back calculation method using twenty-four “known size” populations. Based on the ratio between the predicted and real size, we found four sub-populations ineligible. After excluding the four ineligible sub-populations, the ratio between the estimated size and real size of all populations ranged from 0.54 to 1.44, with the R square of 0.86.

Using the twenty “known size” populations, we back calculated the social network size of the study participants. Overall, the network size of people living in Tbilisi was estimated at 355 (95% (*CI*): 342 ~ 366). Using the male/female and adult ratio of population in Tbilisi, we calculated the social network size of all and adult male and female populations (Table 2).

**Table 2:** The average active social network size of people living in Tbilisi, Georgia 2014.

Sub-population	Active Social Network Size
**Male**	
18–59y	108 [105–112]
Total	154 [148–158]
**Female**	
18–59y	115 [110–118]
Total	201 [194–208]
**Total**	
18–59y	223 [215–230]
Total	355 [342–366]

Numbers in [] are simulation intervals.

### Transparency and popularity bias

In the MSM RDS survey, we estimated the Transparency bias as 26% (95% (*CI*): 23 ~ 29%); equal to a correction factor of 3.83 for NSUM estimates. The Popularity ratio for MSM was estimated at 6.7; which means MSM in Tbilisi had a 6.7 times larger social network size than the general population of Tbilisi.

### Multiplier estimates

Four mobile apps /website multipliers, one service multiplier and one unique object multiplier were used to estimate the MSM population size. The popularity of different mobile apps/website among MSM respondents ranged from 0.7% for the *Hornet* mobile app up to 25.3% for *Gayromeo*. Only 16.8% of MSM utilized the services available to them at the health center. Using differ multipliers, the estimated MSM population size ranged from 988 (unique object) to 22,859 (*Hornet*). The median of all multipliers yielded an estimated size of 4,541 with the acceptable range of 2,700 to 15,809 (Table 3).

**Table 3:** MSM population size estimates in Tbilisi, Georgia using a range of multiplier methods.

Different mobile/web apps; services	Percentage of users			Number whom were counted	Population Size Estimates		
	Point Est.	95% Lower Bound	95% Upper Bound		Point Est.	95% Lower Bound	95% Upper Bound
*Grindr* mobile app	4.1%	1.3%	6.9%	394	9,636	5,701	31,097
*Mamba* web app	10.4%	2.7%	18.1%	611	5,881	3,372	22,961
*Hornet* mobile app	0.7%	0.7%	1.4%	162	22,859	11,362	22,859
*Gayromeo* web app	25.3%	10.7%	39.9%	809	3,201	2,029	7,589
Service use	16.8%	3.8%	29.8%	333	1,980	1,116	8,759
Unique objects	9.7%	3.6%	15.8%	96	988	607	2,648
**Median of all**					4,541	2,700	15,809

### Network based Capture-Recapture

Using the four-identifier categorical variables and the telefunken code, we identified 36 matches between the RDS participants’ telefunken records (205) and recaptured telefunken (770) records. This led to a calculated population size of 4,385 (95% (*CI*): 3,115 ~ 5,654). Using the marginal distribution of the variable used to define the unique identifiers very small expected false match case was seen (0.17) which was discounted due to the likely small effect.

## Discussion

Using different population size estimation methods it has been calculated that the MSM population within Tbilisi is between 0.9% and 2.53% of adult males; this corresponds to 5,100 (95% (*CI*): 3,243 ~ 9,088) men. Taking into account the estimate of HIV prevalence among MSM, reported in 2012 Tbilisi study (RDS 2012) as 13% (95% (*CI*): 8.5 ~ 18.7%), it is estimated that there are 663 (95% (*CI*): 434 ~ 954) MSM infected with HIV. This estimate demonstrate the need to ensure that MSM infected with HIV are identified and supported, linking these potentially marginalized and vulnerable members of the community to appropriate treatment services and further reduce the transmission of HIV infection in the MSM community.

Literature search results through the Internet revealed that there is a lack of publications on population size estimation among MSM across EECA region. Only Serbia has documented the experience and highlighted how the estimates of key populations can be achieved. The majority of the estimates comes from country reports. Our review of 29 country national Global AIDS Response Progress Reports (GARPR) of EECA region showed that population size estimates for MSM have only been estimated for six countries (Azerbaijan, Bosnia and Herzegovina, Moldova, Serbia, Ukraine, Kazakhstan); however, the GARPR reports often do not include details of the PSE methodology, which makes it difficult to assess the credibility of the estimates and also compare the findings. Sometimes, the results are presented as crude number of MSM, not a percentage of MSM in the adult male population, which again presents difficulties in comparing results across countries. In comparison with global and regional estimates for the proportion of MSM, the estimate for Tbilisi, Georgia estimate is comparable to Ukraine, where MSM estimated as 1.7% of the male population (15–59 years), based on NSUM and multiplier methods. The estimated population size of MSM have been reported in a number of other countries including Azerbaijan (0.84–1.68%), Bosnia and Herzegovina (0.4–0.8%), Moldova (1.29%), Serbia (1.4–5.9%), Kazakhstan (1.9% or 3.2%), and Kyrgyzstan (1.08%), also UNAIDS suggest a proportion between 2–5% for EECA, all of which are within the range for the estimate for Tbilisi [21] (See Table 4).

**Table 4:** MSM population size estimates in EECA countries [22–31].

Country	Geographic unit	MSM PSE		Source of the data	References
		Estimated MSM size	Methodology		
Azerbaijan	3 cities (Baku, Ganja, Sumgait), 2011	6,572 (4,396–8,748) 1.26%*(0.84% -1.68%*)	NR	National report	GARPR 2014
Bosnia and Herzegovina	National, 2012	6,900 (4,300–9,500) 0.6%* (0.4%–0.8%*)	NR	National report	GARPR 2014
Moldova	National, 2012	13,500 or (1.29%*)	NR	National report	GARPR 2014
Serbia	National, 2009	20,789–90,104 (1.4%–5.9%)	NR	National report	GARPR 2014
Serbia	National, 2009	20,789–90,104 (1.4%–5.9%)	Multiplier, benchmark methods, capture-recapture	Publication	Comiskey C. et al., Injecting drug users, sex workers and men who have sex with men: a national cross-sectional study to develop a framework and prevalence estimates for national HIV/AIDS programs in the Republic of Serbia. BMJ Open. 2013
Ukraine	National, 2009	95,000–213,000 (1.3%–1.7%)	NSUM, Multiplier	Analytical Report	V. Paniotto, T. Petrenko, V. Kupriyanov, O. Pakhak “Estimating of the Size of Populations with High Risk for HIV, Using the Network Scale-up Method, 2009
Ukraine	National, Urban places, 2012	225,000 (1.7%)	Multiplier	Analytical report	G. Berleva, K. Dumchev, M. Kasianchuk, M. Nikolko, T. Saliuk, I. Shvab, O. Yaremenko “Estimation of the Size of Populations Most-at-Risk for HIV Infection in Ukraine” as of 2012 based on the results of 2011 survey
Kazakhstan	9 regions, 2013	28,840 (1.9%*)	NR	National report	GARPR 2014
Kazakhstan	National, 2013 Astana, 2013 Almaty, 2013 Pavlodar, 2013 Shymkent, 2013	166,073* (3.20%) 5,919* (2.40%) 20,146* (4.50%) 866* (0.90%) 4,322* (2.10%)	Capture-Recapture, NSUM, Wisdom of the Crowds	Abstract	2013 Annual conference -Estimation of the Population Size of Men who have Sex with Men (MSM) in Kazakhstan: Implications for HIV Testing and Surveillance
Kyrgyzstan	National, 2010	17,500 (1.08%*)	NR	Personal contact	

NR–Not Reported; GARPR—Global AIDS Response Progress Reporting

* Calculated by authors, based on country demographic profiles attained from indexmundi.com and citypopulation.de.

We used the median to arrive the estimate out of seven different estimates (with the different lower and upper boundaries), as it provides a more robust estimate as it is not influenced by extreme / outliers and skewed estimates [32–34].

Given the estimated number of MSM in the Tbilisi adult male population and the increasing trend of HIV prevalence among MSM, clearly more needs to be done to identify and link such vulnerable key populations into treatment services and also further reduce the transmission of HIV infection in their community [35;36]. It is critical to understand that different MSM sub-populations could not be reached with the standard HIV preventive package due to different factors including stigma, homophobia and fear of public exposure. In Georgia at present, successful contact with MSM is defined by yearly access to a preventative package, which includes as condoms, lubricant, health information material and counseling about HIV/AIDS. Whilst distribution of condoms and lubricants is an immediate and effective strategy for the general MSM population, it may not reach a number of important sub-groups within this population. MSM who do not gather in communities or who do not identify or disclose their sexual behavior with others will be impossible to reach with an intervention that solely relies on condom/lubricant distribution and counseling. Alternatively, these populations could be targeted with innovative context tailored interventions, e.g. Internet / mobile application based interventions, which have been demonstrated to be popular among MSM; however estimation of coverage of such interventions still remains challenging.

There is a global/regional need to strengthen the capacity and willingness to estimate the population size of MSM; dissemination of our study findings could encourage other countries to implement such studies and also transparently share their results.

Among the different methods that we applied, wisdom of the Crowds was relatively a new method. The estimates that provided by this method had the biggest range and maximum estimate of the MSM size. This also has been reported in the study of PSE of MSM in Ghana [37]. The wide range could be due to misinterpretation of the question by some participants whom have reported their own personal network, rather than the overall size of MSM community in Tbilisi. Others might have reported a huge unbelievable number of MSM as their own desire to show that such behaviors (having sexual contact with another men) are not anymore uncommon. Tbilisi is a big capital city, and MSM might not know or contact with the whole community of MSM living in this big cosmopolitan area. This method may provides more accurate estimates when the member of the community of target population, e.g. MSM, are visible to each other, have gatherings and social events and connected as one solid community. It also provides more precise estimates with bigger sample size; which was not the case for our study. While in MSM population size estimation study in Nairobi, Kenya [15], WOC produced the lowest plausible estimates; In contrast, in our study, WOC method yielded the high estimates. This telling us that the direction of bias using WOC is not predictable. However, since the estimated number of WOC was in range with estimates from other method (some of the multipliers), we decided to include this in the overall population size estimation of MSM; as presented in the result, even if we would have excluded the WOC estimates from the combined overall estimates, the overall size of MSM decreased, but not that much, an ensuring finding that the median is a robust estimator.

This may lead to a wide range of responses and so least robust estimates. This method can be improved by making the question more specific to whom the estimates refers, limit the geographical area which the question is asking about, follow-up questions to ensure that the respondent have understood the question correctly, as well as train the interviewers to ask the question in the same way for all participants.

To provide precise estimates in a population size estimation exercise, with multiple methods and two sources of data (one a general survey and the other an RDS survey among MSM), calculating the sample size is challenging. This is because the shape of the sampling distribution is unknown and the standard error has no parametric closed-form equation. As explained in the method section, we calculated the sample size for estimating the social network size of general and MSM populations. As expected, such sample size was sufficient enough to estimate the size of MSM with acceptable precision for the network scale-up method. The estimates form other methods like multipliers, Handcock, Capture-Recapture and Wisdom of Crowds were less robust and had a wide range. In the literature there is not much about the sample size calculation for a PSE exercise using mix methods. In complex experiments, when multiple explanatory variables are thought to be important, or when non response and missing are likely to occur, simulation approach are recommended for sample size calculation [38]. Such approach can be adopted for PSE studies also.

We would like to acknowledge some of our study’s limitations. Estimating the size of any hidden or hard-to-reach population is a challenge. Although the estimates were robust and have been validated by key stakeholders, they of course have some inherent limitations and cautions; first, size estimation exercises generally cannot estimate the proportion of MSM who are truly hidden and/or MSM who do not even acknowledge that they are MSM. These MSM may not be counted in any data source, including data collected through this study. Secondly, this study was limited to MSM 18 years and older and therefore these estimates do not include MSM younger than 18. Given this, these estimates are likely an underestimation of the MSM population size in Tbilisi, Georgia. Thirdly, adult male population denominators are based on 2002 Census projections by the National Statistics Office and actual census numbers may vary from projections and thus would influence the estimates. Fourth, the quality of the estimate derived from the multiplier method using the website and mobile applications are only as good as the quality of data that was used to produce such estimates. And finally, the accuracy of NSUM estimates is dependent on the accuracy of responses received from the study participants, the quality of the data source for the known population sizes, the transparency of MSM behaviors among the social networks and the random mixing of MSM in the community. In an attempt to reduce bias the analysis has included adjustments for some biases (transparency and popularity), while other biases are harder to measure.

## Conclusions

In conclusion, this is the first population size estimation study among MSM using multiple scientifically acknowledged methods conducted in Tbilisi, Georgia. Our estimates are in line with the current limited estimates available on PSE in EECA countries. Since there is a rising trend of HIV prevalence among MSM, strengthening prevention services to make them more accessible and improved utilization by MSM is an urgent action and should be prioritized in the national strategic program against HIV in Georgia.
